# ToppMiR: ranking microRNAs and their mRNA targets based on biological functions and context

**DOI:** 10.1093/nar/gku409

**Published:** 2014-05-14

**Authors:** Chao Wu, Eric E. Bardes, Anil G. Jegga, Bruce J. Aronow

**Affiliations:** 1 Department of Computer Science, University of Cincinnati, Cincinnati, OH 45221, USA; 2 Division of Biomedical Informatics, Cincinnati Children's Hospital Medical Center, Cincinnati, OH 45229, USA; 3 Department of Pediatrics, University of Cincinnati, Cincinnati, OH 45221, USA

## Abstract

Identifying functionally significant microRNAs (miRs) and their correspondingly most important messenger RNA targets (mRNAs) in specific biological contexts is a critical task to improve our understanding of molecular mechanisms underlying organismal development, physiology and disease. However, current miR–mRNA target prediction platforms rank miR targets based on estimated strength of physical interactions and lack the ability to rank interactants as a function of their potential to impact a given biological system. To address this, we have developed ToppMiR (http://toppmir.cchmc.org), a web-based analytical workbench that allows miRs and mRNAs to be co-analyzed via biologically centered approaches in which gene function associated annotations are used to train a machine learning-based analysis engine. ToppMiR learns about biological contexts based on gene associated information from expression data or from a user-specified set of genes that relate to context-relevant knowledge or hypotheses. Within the biological framework established by the genes in the training set, its associated information content is then used to calculate a features association matrix composed of biological functions, protein interactions and other features. This scoring matrix is then used to jointly rank both the test/candidate miRs and mRNAs. Results of these analyses are provided as downloadable tables or network file formats usable in Cytoscape.

## INTRODUCTION

In order to predict the impact of microRNAs (miRs) on biological systems, it is critical that there is consideration of not only expression levels, differential regulation and strength of interaction with messenger RNA (mRNA) targets, but also the relative importance of those targets in a given biological context. While most miR–mRNA target analyses address the relative accuracy of individual miR target prediction algorithms, less is known regarding how specific biological contexts and functions dictate the relative impact that differentially expressed miRs have on a biological system. Since most miR-ranking approaches against targets have been based on the magnitude by which their target mRNAs are likely to be degraded or inhibited, this approach ignores the possibility that strong mRNA transcriptional control has also affected target gene expression, and this leads to a lack of consideration of important miR target mRNAs among transcriptionally activated genes. To evaluate miRs in a biosystems context, several computational approaches have been developed to identify and prioritize miR–mRNA interactions (1–3). Most of these approaches combine the mRNA and miR expression profiles and identify potential functional miR–mRNA interactions based on the assumption of anti-correlation between a miR and its predicted target mRNA expression levels (e.g. MAGIA (4) and miRGator (3)). Most of the current approaches for ranking miR–mRNA relationships do not leverage the mRNA expression-based functional enrichment data (e.g. enriched biologically processes or pathways of differentially expressed mRNAs). Further, anti-correlation between miRs and mRNAs may not always mean that there is a direct interaction between them. Conversely, coexpressed miR and mRNA could be functionally related. A few of the recent approaches attempt to address these issues. For instance, Suzuki *et al.* developed an approach called GFA (GSEA-FAME analysis) to rank the most significant miRs in cancer transcriptomes based on differential enrichment by the number of miR targets (5). Bryan *et al.* proposed to apply biclustering algorithms to visualize functional miR–mRNA modules (6). Likewise, Li *et al.* used functional annotations (including GeneOntology and Pathway) to prioritize all possible target sites of each miRNA (7), the philosophy of which is fully in accord with what we have sought to enable. 

To address the complexities associated with evaluating and predicting the functional impact of multiple miRs on biological networks, we developed ToppMiR, a web-based analytical system. ToppMiR analyzes and ranks miRs and their putative mRNA targets within either user-defined or transcriptome-profiled biological contexts, and therefore identifies and ranks the potential importance of the miR–mRNA interaction. ToppMiR learns intrinsic and hidden knowledge from the context by recognizing significant features of the gene sets. The mRNA or gene ranking (target and non-target genes) is based on previously published ToppGene and ToppNet (8). Additionally, ToppMiR also ranks the miRs integrating the target predictions (compiled from several different prediction algorithms) and their putative targets’ relative importance in the context. Users can optionally use expression profiles to refine the miR–mRNA interactions and prioritization. ToppMiR further enables extraction and export of either entire or partial networks of miRs, genes and annotations under analysis in a variety of formats (e.g. Cytoscape (9) and Gephi (10)) to facilitate further analyses.

## MATERIALS AND METHODS

**Figure 1.**
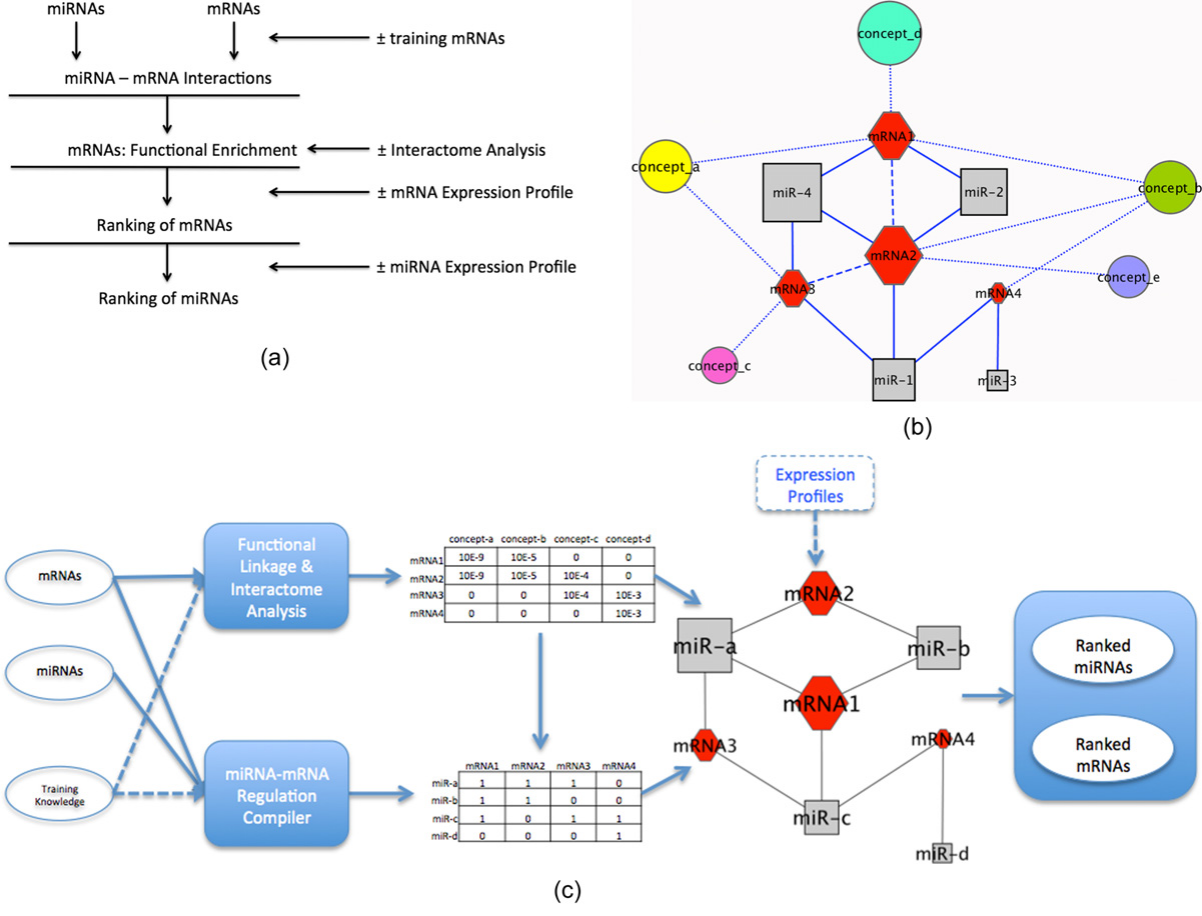
(**a**) Layered representation of ToppMiR workflow. Arrows in the figure indicate the flow of the pipeline and ± represents optional input or analytical steps specified by users. (**b**) Schematic representation of ToppMiR workflow. Arrows indicate the flow of the application while a dashed arrow indicates an optional input. After inputs of lists of miRs and mRNAs, ToppMiR identifies regulations between them and then prioritize the mRNAs based on their enriched terms and relative importance in the Interactome compared to the training set if applicable. Following these, miRs will be prioritized based on their connectivities to their mRNA targets. Expression profiles are optional to facilitate the prioritization. (**c**) A network demonstration on miRs, target mRNAs and biological concepts. Each circle represents a biological concept, each gray rectangle represents a microRNA while each red hexagon represents a mRNA. A solid line indicates a miR–mRNA regulation, a dotted line indicates a mRNA–concept association and a dashed line indicates a protein–protein interaction. The sizes of the nodes reflect their relative functional significance.

ToppMiR's approach to miR/mRNA prioritization can be summarized as follows: annotations retrieved from the gene set enrichment analysis are ranked based upon their nominal *P* values, mRNA targets are ranked based upon their connectivity to annotations and the PPI analysis if applicable (i.e. a concrete training profile is present), and finally candidates are ranked based on their connectivity to their target mRNAs (Figure 1a and b). Thus, an mRNA target associated with more significant annotation concepts will be prioritized higher, as will be a miR that interacts with more significant mRNA targets. A demonstration of this is shown in Figure 1c where a solid line indicates a putative miR–mRNA interaction, a dashed line indicates a protein–protein interaction, and a dotted line represents a mRNA–concept association. A training set of genes is optional in the analysis pipeline. If a user wishes to define a given biological context, this is done by providing to ToppMir a list of specific genes with known functional significance—‘training genes’. The training genes are then used to facilitate the prioritization of the test set of genes.

### Compilation of miR target predictions

ToppMiR uses miR target predictions from seven different sources (PicTar (11–14), mirSVR (15,16), TargetScan (17–20), MSigDB (21,22) and PITA (23)) including experimentally verified miR targets (miRecords (24) and miRTarbase (25)). Since the overlaps among the target prediction algorithms are low (26) (see miR-9 example in Supplementary file), we use the union of all these predictions as candidate miR–mRNA interactions whose significance can be subsequently evaluated.

### Gene set functional enrichment

ToppMiR adopts the approaches of gene set functional enrichment analysis from ToppGene Suite which applies Hypergeometric distribution with Bonferroni or False Discovery Rate (FDR) correction to determine the statistical significance of the annotations.

### Interactome analysis

For the analysis of mRNAs on *Interactome* (27), we used HITS (Hyperlink-Induced Topic Search) with priors (28,29). A ‘back probability *β*’, where $0 \le \beta \le
                            1$, is defined as the probability to jump back to the ‘root’ set at each step. Training sets are considered as the ‘root’ sets in the analysis. A range from 0.3 to 0.5 was recommended for *β* in previous study (8).

### Prioritization of genes in gene-annotation network

**Figure 2.**
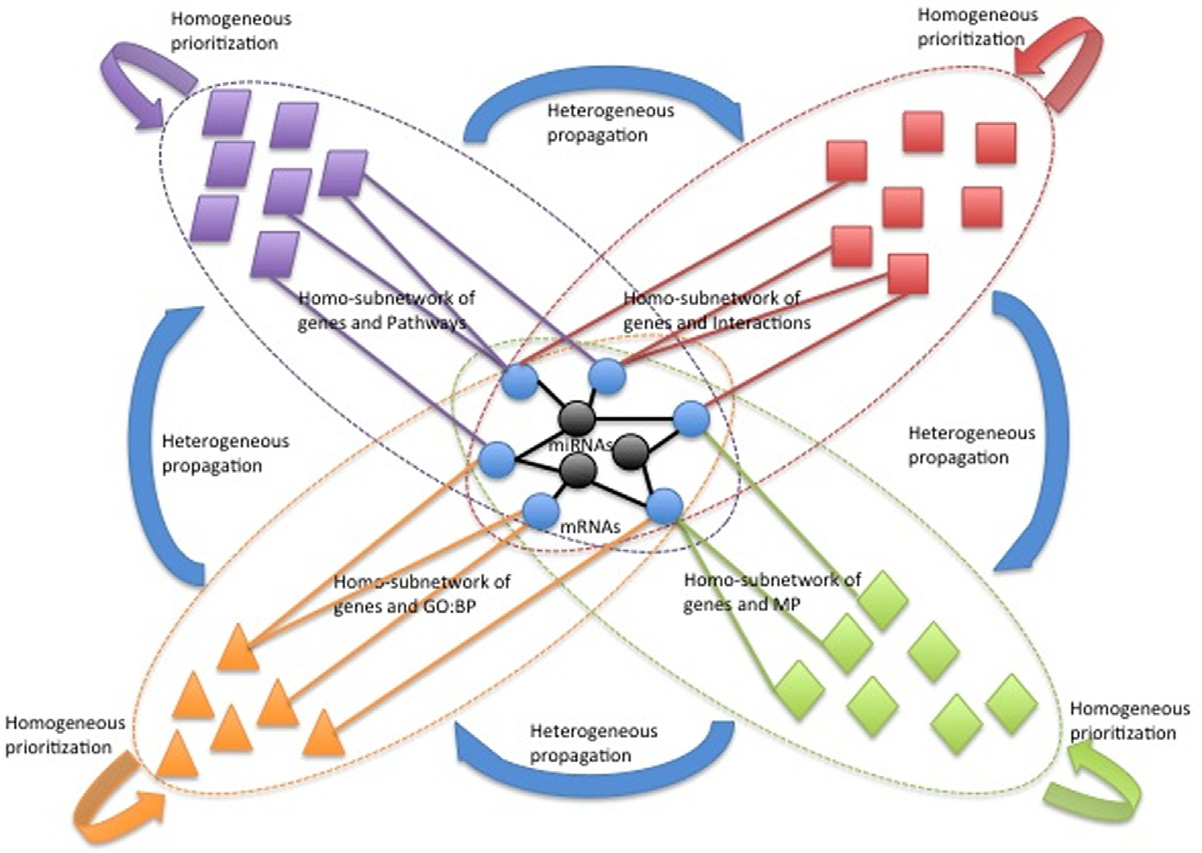
Prioritization of miRs and mRNAs based on heterogeneous network propagation. Blue circles indicate mRNA targets, and black circles indicate miRs. Other nodes represent biological concepts. Concepts from the same categories share the same color. mRNAs will be first prioritized based on their connectivities to important biological concepts of single categories, and then their significance scores will be propagated across categories until they converge.

mRNAs are ranked based on their associations with significant biological concepts. The functional significance of the gene set is statistically evaluated by methods discussed above and the information is represented by enrichment result, which is then used to rank test genes. Specifically, a significant score of a gene of a single category is defined as the sum of the reciprocal *P* values of the biological concepts it is associated with. Following this, the significant score is propagated across heterogeneous categories such as Gene Ontology (GO), phenotypes and biological pathways until it is converged. Following this, the input miRs of interest are prioritized upon the ranking of their putative mRNA targets. The overall ranking strategy is illustrated in Figure 2 and the detailed ranking strategy can be found in the supplementary file.

### Prioritization of miRs by expression and biological functional relevance

**Figure 3.**
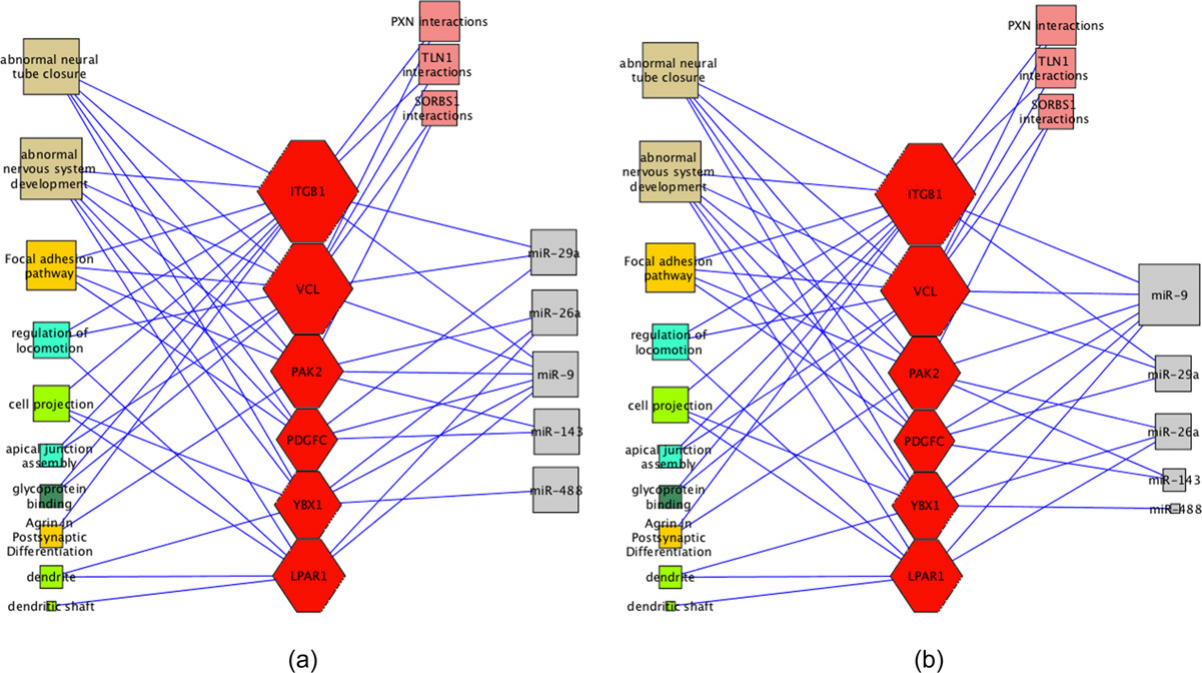
(**a**) Before miRNA prioritization. Rectangles on the leftmost are biological concepts, red hexagons in the middle are mRNA targets, pink rectangles are protein–protein interactions and gray rectangles on the rightmost are miRs. Sizes of the biological concepts and mRNA targets indicate their relative functional relevance. Edges indicate associations/regulations between the nodes. (**b**) After miRNA prioritization. Sizes of the miRNAs reflect their centrality to the important mRNA targets.

After the analysis and prioritization of mRNAs, ToppMiR ranks the miRs based on their putative regulations to their target mRNAs. To do this, a significance score ${\rm Sig}_{{\rm miR}}$ is assigned to each miR that is the sum of the significance of all its target mRNAs. Figure 3 demonstrates this approach by presenting two concept–mRNA–miR networks: (a) before and (b) after miRs prioritization. The change in size and position of the miR nodes indicates their significance scores from the ranking algorithm.

ToppMiR also enables a user-specified coefficient *α* to act as a cutoff value that defines which mRNAs are most significant for evaluation of their connectivity to a list of miRs. The intent is to focus on mRNA targets that have the most significance for the biological system as defined by the enriched features of the user-specified training set or the test set gene list. By our experiments on the validation of miR–mRNA pairs derived from PubMed, we observed that a cutoff value of 40% usually generates the best performance. Thus, a default coefficient is set to this value denoted as *α* in Equations (1) and (2). Overall, if we let G denote the final ranked gene list, the significance score of the miR can be calculated as

(1) \begin{equation*} {\rm Sig}_{{\rm miR}} = \sum\nolimits_{i =
                            1}^{\alpha \cdot \left| {{\rm G}_{{\rm test}} } \right|} {{\rm
                            Sig}_{\left( i \right){\rm mRNA}\,{\rm target}} }
                            \end{equation*}

By the use of the different G (test, training), ToppMiR allows users to choose whether the miR ranking is analyzed based on either the test or training set features. If the user chooses to prioritize miRs based on both sets, the analysis is done in a similar manner compared to only using their target mRNAs in the training set. An extra step will take place to multiply the significance score of each miR by the sum of significance score of its target mRNAs in the training set; therefore, a significance score of the miR can be interpreted as

(2) \begin{eqnarray*}
                            &&{\rm Sig}_{{\rm miR}} = \nonumber
                            \\&&\quad \sum {{\rm Sig}_{{\rm mRNA}\,{\rm target}}
                            {\rm G}_{{\rm train}} } \cdot \sum\nolimits_{i = 1}^{\alpha \cdot \left|
                            {{\rm G}_{{\rm test}} } \right|} {{\rm Sig}_{\left( i \right){\rm
                            mRNA}\,{\rm target}} }
                        \end{eqnarray*}

### Integrating analyses in Euclidean space

When expression profiles are available, ToppMiR can take advantage of such information by integrating enrichment analysis result and expression profiles together in Euclidean space. The motivation is to comprehend the significance of each mRNA and/or miR from multiple aspects. ToppMiR accepts text input of mRNA and/or miR expression profiles under a required format explained on the corresponding input page of ToppMiR application online. ToppMiR accepts HGNC symbol or Entrez ID for the identifier for the mRNAs and miRs when uploading a text file. The other two columns can be specified as ‘Expression level’ and/or ‘Fold change’. Thus an overall vector profile in multidimensional Euclidean space for each mRNA or miR can be calculated as

(3) \begin{eqnarray*}&&{\rm Sig}_{{\rm overall}} =
                            \nonumber \\&&\quad \big\| {{\rm Sig}_{{\rm
                            enrichment}\,{\rm analysis}}} + {\rm Sig}_{{\rm expression}\,{\rm
                            level}} + {\rm Sig}_{{\rm fold}\,{\rm change}}
                            \big\|\end{eqnarray*}

### Implementation and user access

ToppMiR has been implemented as a web-accessible system using Java that runs across a cluster of Linux servers utilizing a Sun Glassfish Enterprise Server environment. ToppMiR requests ToppGene functions via Java Messaging Services (JMS). JMS allocates gene-list enrichment jobs and protein–protein interaction analysis jobs to available ToppGene resources through a load balancer. ToppMiR generates and visualizes network-based data using JUNG libraries (30) and provides the option to download tab-separated text files or GML format files compatible with Cytoscape (9). ToppMiR is publicly available at http://ToppMiR.cchmc.org.

## RESULTS

The performance of ToppMiR was evaluated based on two types of comparisons: large-scale cross-validations and small-scale test cases. For large-scale cross-test set/validation set analyses, we used PubMed abstracts that co-cite specific miR and mRNAs or pSILAC data sets, while for small-scale test cases we used tissue-specific miRs whose expression was shown to be altered using qPCR data. miR ranking was performed using both the scenarios—training set dependent and independent. We used expression profiles to further refine the candidate genes and miR rankings where possible. Using these series of test cases, we also demonstrate the utility of ToppMiR in knowledge discovery and novel hypothesis generation. Additional details of validation methods and their application to different test scenarios can be found in the supplementary data.

### ToppMiR validation using miRNA–mRNA PubMed co-citation

We developed a series of validation tests based on manual PubMed searches using a set of publications that were published between October and December 2011. These articles reported at least one experimentally validated miR–mRNA interaction of functional significance but were not present in Toppgene's database (February 2012). The validation set we developed comprised 16 pairs of novel miR–mRNA pairs at that time. We used this data to exemplify the capability of ToppMiR to prioritize those interactions relative to a random set of miR targets and thus demonstrate its potential to rank highly biologically significant miR–mRNA interactions. Additionally, to investigate the effects of training sets on ToppMiR ranking, we performed the prioritization experiments in both training set-dependent and independent scenarios. Appropriate training sets were manually compiled depending on the biological instance reported in each of the 14 selected publications. For instance, for a publication reporting either a tumor suppressor miR or an oncomiR (31), we used cancer-related genes from the Cancer Gene Census database (32) as the training set. Similarly, for a publication that reported the role of miR-106b in impaired cholesterol efflux (33), we applied known genes associated with the GO term ‘cholesterol efflux’ (GO:0033344) as the training set.

For each of the experiments, we mixed the ‘target’ miR (from the publication) with randomly selected 19 miRs to comprise the set of 20 miRs for prioritization task. In case of target mRNAs (genes), we added 99 randomly picked genes to the selected miR ‘target’ gene (from the same publication) to make a set of 100 genes for each run. For each miR–mRNA pair, we performed 100 prioritization runs (with and without using training sets). The rank of the selected miR and the target gene in the resulting list, following ToppMiR prioritization, was recorded. Receiver operating characteristic (ROC) curves were plotted based on the sensitivity/specificity values, and area under curve (AUC) was computed as the standard measure of the performance of the method. Sensitivity was defined as the frequency of ‘target’ miRs or genes that are ranked above a particular cutoff, and specificity as the percentage of miRs or genes ranked below the threshold in this case.

Of the 16 pairs of miR–mRNA (32 sets of validation results, 16 each for miR and mRNA), ToppMiR was able to rank the selected miR with an AUC score ≥ 0.8 in 13 out of 16 cases, with an AUC score greater than 0.8 for 11/16 targets. This ranking was performed without using any training set.

We repeated the analyses using appropriate training sets to evaluate the performance of ToppMiR. Interestingly, no significant improvement was observed for the majority of the pairs in terms of the ToppMiR ranking when a training set was used in the analysis. Among the 16 pairs tested, only 3 miRs and 6 mRNA showed an improved performance (see supplementary file).

In our 14 selected publications, 11 were cancer-related while three were non-cancer-related (cholesterol efflux (33), cardiac arrhythmias (34) and submandibular gland branching morphogenesis (35)). ToppMiR was able to prioritize these miR–mRNA interactions with relatively high AUC scores (0.86 on average). Additional details of the PubMed publication validation can be found in the Supplementary Data (Data file S1).

### pSILAC data set validation

Using the experimentally supported targets from the pSILAC (stable isotope labeling by amino acids in cell culture) data set (36,37) we evaluated the performance of ToppMiR ranking of miRs. Briefly, 100 mRNAs that were most differentially expressed when a specific miR was knocked down or overexpressed are selected as ‘true targets’ on a genome-wide scale. The pSILAC data comprise five overexpressed miRs (let-7b, miR-155, miR-1, miR-16 and miR-30) and one knocked-down miR (let-7b). In each validation run, the target mRNAs of a particular miR (with one mRNA removed as the ‘target’) was used as the training set. The ‘target’ mRNA was then mixed with 99 random mRNAs to make a test set of 100 genes to form a training set-dependent scenario. The rank of the ‘target’ mRNA in the resulting list, following ToppMiR prioritization, was recorded. This process was repeated for each gene in the list. Likewise, for ranking the miRs, the ‘target’ miR was mixed with another 19 random miRs to comprise the candidate miRNA list. We repeated this experiment for the top 20 mRNAs of the sorted list of each of the 6 individual data sets, thus the total number of experiments was 120. ToppMiR was able to rank the ‘target’ miR among the top 10% 69 times out of 120 (∼58%) and 111 times among the top 20% of the prioritized lists (∼92.5%). A ROC curve was plotted to visualize the result, with an AUC score of 0.93.

**Figure 4.**
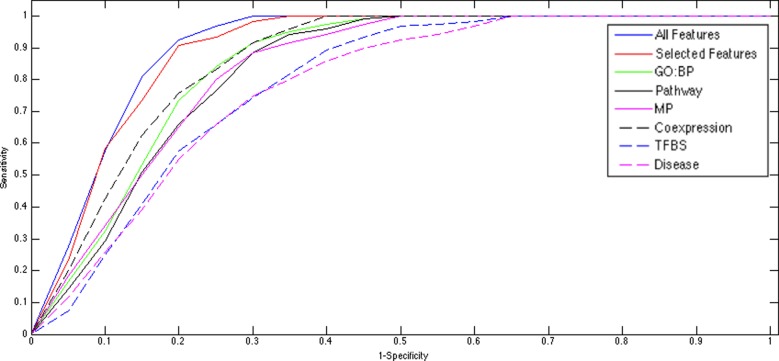
ROC curves of LOOCV on pSILAC data. The blue curve was generated using all features with an AUC of 0.93. The red curve was generated using ‘core’ features of GO: BP, Pathway, Mouse Phenotype and Coexpression with an AUC of 0.92. Other curves were generated using single features respectively as listed in the legend.

To examine the effectiveness of different categories of annotations, we also ran the experiments with the same sets of miRs and mRNAs in different settings of annotation features, including GO: Biological Process (BP), Mouse Phenotype (MP), Pathways, Coexpressions, Transcription Factor Binding Sites and Diseases, respectively, and plotted the ROC curves (Figure 4). Some categories, namely GO: BP, MP, Pathways and Coexpressions, achieved better AUC scores than others, indicating that annotations of these categories could be more informative. This observation concurred with previous studies (7,38). Thus we performed another examination using only these four selected categories in the same setting of miRs and mRNAs. In this experiment, ToppMiR was able to rank the ‘target’ miR 109 times out of 120 among the top 20% of the prioritized lists (∼90.8%) with an overall AUC score of 0.919. The comparable result suggested users could approximate the result generated from using all features by using only some of the ‘core’ categories indicating the power of data integration (see Figure 4).

On the other hand, the overall AUC score of ‘target’ mRNAs was 0.76. Noted the score being much better than most ‘false negative’ genes in the genome, it was lower than that of the ‘target’ miRs. We hypothesize this behavior was attributed to the fact that the correlations between the ‘target’ miRs and the training set of genes were greater than the functional correlations between their putative mRNA targets.

### miR and mRNA expression data integration

Finally, we also evaluated the performance of ToppMiR combining expression profiles of miR and mRNA with interaction and enrichment analysis. To evaluate biologically significant miRs within different biological contexts, we use a miR expression data set from GSE34199 (39) which contains both undifferentiated human embryonic stem cell lines and four normal adult human tissues. miR profiles included 470 miRs as assayed on the Agilent Human miRNA microarray platform. For the test set of mRNA expression, we used BodyMap (40,41), a collection of highly expressed gene profiles in various normal human tissues. Two brain overexpressed miRs, namely miR-9 and miR-124, were selected for ToppMiR ranking. As part of training set, using GO and Mammalian Phenotype Ontology annotations, we compiled three distinct yet overlapping training sets of genes associated with the following essential functions of the brain: memory and learning, neuron development and neuron physiology (see supplementary file).

For each ‘target’ miR and training set of genes, 49 random miRs were selected for each individual run. This process was repeated 100 times for each combination of ‘target’ miR and training set. The ranks of the ‘target’ miR before and after expression profile integration were both recorded. As the results indicated, integrating expression profiles greatly enhanced the performance to identify significant miRs.

Without expression profile data integration, the ranking of miR-124 did not change as a function of alternative training profiles (ranked in the range of 8.2–8.5 out of 50 on average). On the other hand, the estimated impact of miR-9 did not change as a function of neuron development and physiology training profiles (average ranked at 8.8 and 9.2, respectively). However, the use of training genes associated with memory and learning caused ToppMiR to rank miR-9 considerably higher (ranked at 4.2 on average).

## DISCUSSION

ToppMiR seeks to enable discovery and hypothesis generation about the potential impact of miRs within specific biological systems. In this study, our approach to validating the ability of ToppMiR to usefully rank a list of candidate miRs is based on literature miR–mRNA co-citations as a form of gold standard that should rank highly in test comparisons of miRs and mRNAs. In approaching these tests, we constructed several scenarios that use explicit biological system-associated genetic knowledge as well as mRNA and miR expression data from different contexts. The results of these analyses and comparisons have illustrated several different approaches that can lead to potentially interesting biological systems-level predictions of miR functions suitable for experimental validation for their strong impact on the function of a biological system.

By using ranked features and functions associated with a biological context based on a gene training set or the observed pattern of mRNA expression, the relative importance of different miR targets can be evaluated. ToppMiR allows for this and thus provides valuable perspectives for the exploration of the potential functional significance of miRs and their validated or predicted targets. Li *et al.* proposed to use functional annotations to predict and prioritize miR targets, and showed that validated targets exhibited greater significance (7). We derived the framework of ToppMiR following a similar perspective and provided a web tool that generated analysis results on multiple miRs in real-time. In contrast to other miR analysis tools, ToppMiR does not assume that the most relevant mRNA targets are those that decrease upon miR expression increase. Rather it performs analyses that focus on miR–mRNA pair recognition, using enrichment analysis on target mRNAs and the integration of the two to leverage knowledge of a biological system such that the interesting miRs and mRNA targets are identified based on their centrality to the most significant properties of the biological state or system.

In the comparison between scenarios of training set dependent and training set free, we have observed that employing a biologically appropriate training set never deteriorates the performance of the prioritizations. It seems to be especially helpful when the user is interested in exploring the functionality of miRs in a particular biological context that is represented by a selected training set that can encompass groups of genes known to be necessary for the development or function of a given biological state.

Nevertheless, our approach also has some limitations that should be considered in its practical use. From the perspective of candidate miR–mRNA targets, we compiled seven different sources curating validated, observed, and predicted miR–mRNA interaction potential. Whereas miRTarbase and miRecords represent largely validated interactions, the other data sources contain varying degrees of large-scale observational (e.g. mirSVR) or only predicted interactions that may not validate under some circumstances. Most prediction algorithms utilize configurable miR sequence conservation and specificities to generate a likelihood of a conserved miR–mRNA target site (17). While the predictions greatly enlarge the potential impact of less studied miRs, the lack of agreement among these predicted interactions shows diversity even when their individual predictions are done with high confidence (26). Therefore, the risks of false positive interactions cannot be avoided. Another important effect can be driven by a user's hypothesis as to the most important biological features of a given system that leads them to select a given training set. This suggests that some scenarios of prioritizations of genes and miRs may benefit from more sophisticated and contextual approaches to developing training genes. For example, in transiting from stem cell to lineage restricted cell, it may be most important to consider targets that would cause the alternative differentiation pathway rather than the one that is to be formed. An additional layer of complexity can be envisaged based on species-specific evolution of miRs, their regulatory behavior, and the changes in miR recognition sequences in genes' 3′UTRs.

Importantly, we hypothesize new miR-centered computational approaches based on knowledge extraction, large-scale expression pattern analyses, and the effects of disease associated biological processes such as adaptation to polarized environments or different genetic variation and mutation will all have the potential to greatly improve our ability to identify critical miR regulatory relationships. In evaluating factors that led to validated miR targets to be highly ranked, it is clear that the number of miR targets may not be nearly as important as the centrality of the target for an important biological network. In other words, significant target mRNAs are those that are involved in regulating critical biological processes or pathways. Thus, well-designed computational approaches to recognize those mRNAs and that assign them more weight is key factor to optimize. An inspiring future direction is to build miR models that integrate knowledge of the mRNAs that play quantitative roles in the determination of biological states (42) such that a biological system can be analyzed based on dynamic considerations of network function.

We believe that ToppMiR is a unique computational tool capable of improving our ability to predict significant miRs and miR targets in diverse biological contexts. Given what ToppMiR can do, additional contextual community detection technologies can now be applied to identify overlapping and/or non-overlapping functional modules (43).

When prioritizing target mRNAs, we treated mRNA-annotation networks as directional, which allowed us to employ InDegree, SALSA (44) and other alternative algorithms based on random walk models (details in Supplementary file). Directional relationships are critical to incorporate into biological systems modeling and network analysis. Most gene to biology linkages are directional, i.e. most genes are suppressors of diseases and phenotypes; some mutant alleles have dominantly acting effects on disease; miRs are suppressors of gene transcripts. In contrast, transcription factor binding sites, gene coexpression patterns, gene-ontology features, protein interactions, and pathways are positively determined by expressed genes. In order to apply the algorithms, the edges were being treated as bidirectional in our application. While our approaches show lots of promising results, its application and evaluation in a variety of scenarios is now critical to determine when important miRs and miR targets in different biological contexts may be surmised to play critical roles in the determination of systems' function in health and disease.

## SUPPLEMENTARY DATA

Supplementary Data are available at NAR Online.

Supplementary Data

## References

[1] Nam S., Li M., Choi K., Balch C., Kim S., Nephew K.P. (2009). MicroRNA and mRNA integrated analysis (MMIA): a web tool for
                        examining biological functions of microRNA expression.

[2] Mestdagh P., Lefever S., Pattyn F., Ridzon D., Fredlund E., Fieuw A., Ongenaert M., Vermeulen J., De Paepe A., Wong L. (2011). The microRNA body map: dissecting microRNA function through
                        integrative genomics.

[3] Cho S., Jun Y., Lee S., Choi H.-S., Jung S., Jang Y., Park C., Kim S., Lee S., Kim W. (2010). miRGator v2.0: an integrated system for functional investigation
                        of microRNAs.

[4] Bisognin A., Sales G., Coppe A., Bortoluzzi S., Romualdi C. (2012). MAGIA2: from miRNA and genes expression data integrative analysis
                        to microRNA–transcription factor mixed regulatory circuits (2012
                        update). Nucleic Acids Res.

[5] Suzuki H.I., Mihira H., Watabe T., Sugimoto K., Miyazono K. (2012). Widespread inference of weighted microRNA-mediated gene
                        regulation in cancer transcriptome analysis.

[6] Bryan K., Terrile M., Bray I.M., Domingo-Fernandéz R., Watters K.M., Koster J., Versteeg R., Stallings R.L. (2014). Discovery and visualization of miRNA–mRNA functional
                        modules within integrated data using bicluster analysis.

[7] Li J., Zhang Y., Wang Y., Zhang C., Wang Q., Shi X., Li C., Zhang R., Li X. (2014). Functional combination strategy for prioritization of human miRNA
                        target. Gene.

[8] Chen J., Bardes E.E., Aronow B.J., Jegga A.G. (2009). ToppGene Suite for gene list enrichment analysis and candidate
                        gene prioritization. Nucleic Acids Res..

[9] Shannon P., Markiel A., Ozier O., Baliga N.S., Wang J.T., Ramage D., Amin N., Schwikowski B., Ideker T. (2003). Cytoscape: a software environment for integrated models of
                        biomolecular interaction networks. Genome Res..

[10] Bastian M., Heymann S., Jacomy M. (2009). Gephi: an open source software for exploring and manipulating
                        networks.. International AAAI Conference on Weblogs and Social Media.

[11] Krek A., Grun D., Poy M.N., Wolf R., Rosenberg L., Epstein E.J., MacMenamin P., da Piedade I., Gunsalus K.C., Stoffel M. (2005). Combinatorial microRNA target predictions. Nat. Genet..

[12] Grün D., Wang Y.-L., Langenberger D., Gunsalus K.C., Rajewsky N. (2005). microRNA target predictions across seven
                            *Drosophila* species and comparison to mammalian
                        targets. PLoS Comput. Biol..

[13] Lall S., Grun D., Krek A., Chen K., Wang Y.-L., Dewey C.N., Sood P., Colombo T., Bray N., MacMenamin P. (2006). A genome-wide map of conserved microRNA targets in C.
                        elegans. Curr. Biol..

[14] Chen K., Rajewsky N. (2006). Natural selection on human microRNA binding sites inferred from
                        SNP data. Nat. Genet..

[15] Betel D., Koppal A., Agius P., Sander C., Leslie C. (2010). Comprehensive modeling of microRNA targets predicts functional
                        non-conserved and non-canonical sites. Genome Biol..

[16] Betel D., Wilson M., Gabow A., Marks D.S., Sander C. (2008). The microRNA.org resource: targets and expression. Nucleic Acids Res..

[17] Lewis B.P., Burge C.B., Bartel D.P. (2005). Conserved seed pairing, often flanked by adenosines, indicates
                        that thousands of human genes are microRNA targets. Cell.

[18] Friedman RC, Farh KK, Burge CB, Bartel DP (2009). Most mammalian mRNAs are conserved targets of
                        microRNAs. Genome Res..

[19] Grimson A., Farh K.K.-H., Johnston W.K., Garrett-Engele P., Lim L.P., Bartel D.P. (2007). MicroRNA targeting specificity in mammals: determinants beyond
                        seed pairing. Mol. Cell.

[20] Garcia D.M., Baek D., Shin C., Bell G.W., Grimson A., Bartel D.P. (2011). Weak seed-pairing stability and high target-site abundance
                        decrease the proficiency of lsy-6 and other microRNAs. Nat. Struct. Mol. Biol..

[21] Subramanian A., Tamayo P., Mootha V.K., Mukherjee S., Ebert B.L., Gillette M.A., Paulovich A., Pomeroy S.L., Golub T.R., Lander E.S. (2005). Gene set enrichment analysis: a knowledge-based approach for
                        interpreting genome-wide expression profiles. Proc. Natl. Acad. Sci. U.S.A..

[22] Liberzon A., Subramanian A., Pinchback R., Thorvaldsdóttir H., Tamayo P., Mesirov J.P. (2011). Molecular signatures database (MSigDB) 3.0. Bioinformatics.

[23] Kertesz M., Iovino N., Unnerstall U., Gaul U., Segal E. (2007). The role of site accessibility in microRNA target
                        recognition. Nat. Genet..

[24] Xiao F., Zuo Z., Cai G., Kang S., Gao X., Li T. (2009). miRecords: an integrated resource for microRNA–target
                        interactions. Nucleic Acids Res..

[25] Hsu S.-D., Lin F.-M., Wu W.-Y., Liang C., Huang W.-C., Chan W.-L., Tsai W.-T., Chen G.-Z., Lee C.-J., Chiu C.-M. (2011). miRTarBase: a database curates experimentally validated
                        microRNA–target interactions. Nucleic Acids Res..

[26] Sethupathy P., Megraw M., Hatzigeorgiou A.G. (2006). A guide through present computational approaches for the
                        identification of mammalian microRNA targets. Nat. Methods.

[27] Maglott D., Ostell J., Pruitt K.D., Tatusova T. (2011). Entrez Gene: gene-centered information at NCBI. Nucleic Acids Res..

[28] Kleinberg J.M. (1999). Authoritative sources in a hyperlinked
                        environment. J. ACM.

[29] White S., Smyth P. (2003). Proceedings of the ninth ACM SIGKDD International Conference on
                        Knowledge Discovery and Data Mining.

[30] Madadhain J., Fisher D., Smyth P., Boey Y. (2005). Analysis and visualization of network data using
                        JUNG. J. Stat. Softw..

[31] Zheng B., Liang L., Huang S., Zha R., Liu L., Jia D., Tian Q., Wang Q., Wang C., Long Z. (2011). MicroRNA-409 suppresses tumour cell invasion and metastasis by
                        directly targeting radixin in gastric cancers.

[32] Futreal P.A., Coin L., Marshall M., Down T., Hubbard T., Wooster R., Rahman N., Stratton M.R. (2004). A census of human cancer genes. Nat. Rev. Cancer.

[33] Kim J., Yoon H., Ramírez C.M., Lee S.-M., Hoe H.-S., Fernández-Hernando C., Kim J. miR-106b impairs cholesterol efflux and increases Aβ
                        levels by repressing ABCA1 expression.

[34] Yang B., Lin H., Xiao J., Lu Y., Luo X., Li B., Zhang Y., Xu C., Bai Y., Wang H. (2011). The muscle-specific microRNA miR-1 regulates cardiac
                        arrhythmogenic potential by targeting GJA1 and KCNJ2. Nat. Med..

[35] Rebustini I.T., Hayashi T., Reynolds A.D., Dillard M.L., Carpenter E.M., Hoffman M.P. (2012). miR-200c regulates FGFR-dependent epithelial proliferation via
                        Vldlr during submandibular gland branching morphogenesis. Development.

[36] Schwanhäusser B., Gossen M., Dittmar G., Selbach M. (2009). Global analysis of cellular protein translation by pulsed
                        SILAC. Proteomics.

[37] Selbach M., Schwanhausser B., Thierfelder N., Fang Z., Khanin R., Rajewsky N. (2008). Widespread changes in protein synthesis induced by
                        microRNAs. Nature.

[38] Chen J., Xu H., Aronow B., Jegga A. (2007). Improved human disease candidate gene prioritization using mouse
                        phenotype. BMC Bioinformatics.

[39] Mallon B.S., Chenoweth J.G., Johnson K.R., Hamilton R.S., Tesar P.J., Yavatkar A.S., Tyson L.J., Park K., Chen K.G., Fann Y.C. (2013). StemCellDB: the human pluripotent stem cell database at the
                        National Institutes of Health. Stem Cell Res..

[40] Kawamoto S., Yoshii J., Mizuno K., Ito K., Miyamoto Y., Ohnishi T., Matoba R., Hori N., Matsumoto Y., Okumura T. (2000). BodyMap: a collection of 3′ ESTs for analysis of human
                        gene expression information. Genome Res..

[41] Sese J., Nikaidou H., Kawamoto S., Minesaki Y., Morishita S., Okubo K. (2001). BodyMap incorporated PCR-based expression profiling data and a
                        gene ranking system. Nucleic Acids Res..

[42] Jiang Q., Hao Y., Wang G., Juan L., Zhang T., Teng M., Liu Y., Wang Y. (2010). Prioritization of disease microRNAs through a human
                        phenome-microRNAome network. BMC Syst. Biol..

[43] Palla G., Derenyi I., Farkas I., Vicsek T. (2005). Uncovering the overlapping community structure of complex
                        networks in nature and society. Nature.

[44] Lempel R., Moran S. (2000). SALSA: the stochastic approach for link-structure
                        analysis..

