# Supplementary material for: ToppMiR: ranking microRNAs and their mRNA targets based on biological functions and context
Source: Nucleic Acids Res. 2014 May 14;42(Web Server issue):W107–13. doi: 10.1093/nar/gku409 (PMC4086116; doi:10.1093/nar/gku409)
Supplement: Supplementary Data [file supp_42_W1_W107__index.html]

Supplementary Data 

# ToppMiR: ranking microRNAs and their mRNA targets based on biological functions and context

## Supplementary Data

**Files in this Data Supplement:**

- SUPPLEMENTARY DATA
- SUPPLEMENTARY DATA
- SUPPLEMENTARY DATA
- SUPPLEMENTARY DATA
- SUPPLEMENTARY DATA
- SUPPLEMENTARY DATA
