# Supplementary figures and images for: ToppMiR: ranking microRNAs and their mRNA targets based on biological functions and context
Source: Nucleic Acids Res. 2014 May 14;42(Web Server issue):W107–13. doi: 10.1093/nar/gku409 (PMC4086116; doi:10.1093/nar/gku409)

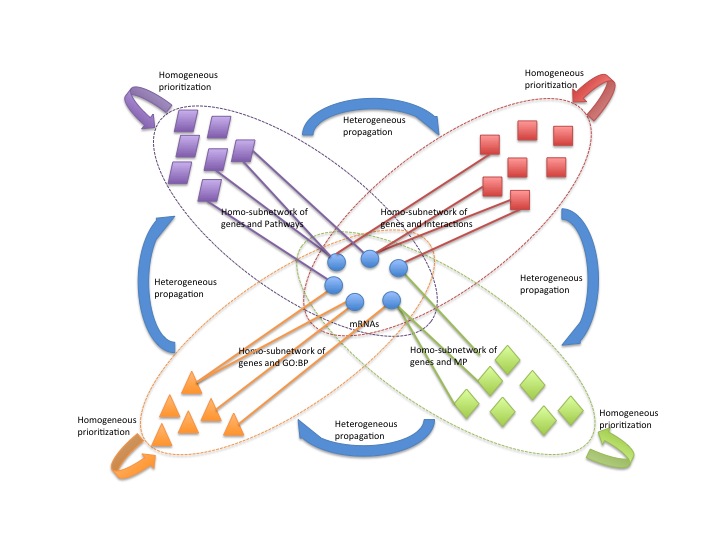

Supplement: Supplementary Data [file supp_gku409_nar-00593-web-b-2014-File010.jpg]

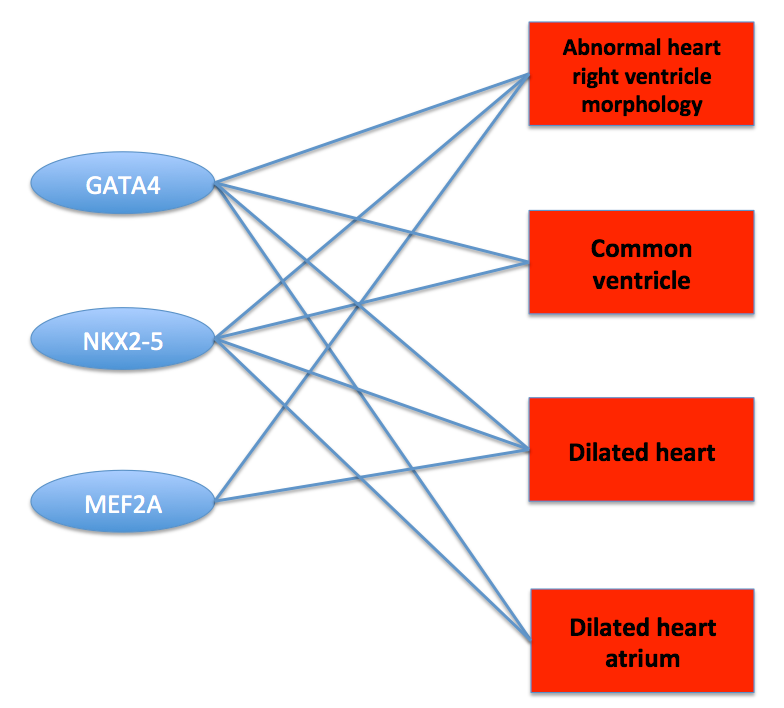

Supplement: Supplementary Data [file supp_gku409_nar-00593-web-b-2014-File009.png]
