# Supplementary material for: ToppMiR: ranking microRNAs and their mRNA targets based on biological functions and context
Source: Nucleic Acids Res. 2014 May 14;42(Web Server issue):W107–13. doi: 10.1093/nar/gku409 (PMC4086116; doi:10.1093/nar/gku409)
Supplement: Supplementary Data [file supp_gku409_nar-00593-web-b-2014-File013.docx]

**Prioritization of mRNAs in gene-annotation network**

ToppMiR prioritizes mRNAs (targets and non-targets of miRs) in gene-annotation network where annotations are generated from gene set functional enrichment analysis. Therefore, genes may be associated with biological annotations from various and independent categories including Gene Ontology, Mouse Phenotypes, Biological Pathways and etc. Genes and biological annotations from each individual category exhibit a homogeneous bipartite (1). Fig.S1 demonstrates such a bipartite where blue ovals representing genes and red rectangles representing phenotype terms. Therefore, mRNAs and annotations of different categories form a heterogeneous as demonstrated in Fig.S2 (inspired by MIN-prop framework ([2](#_ENREF_2))). In the proposed framework, ToppMiR first prioritizes genes in local homogeneous networks comprised of genes and biological annotations of a single category by random walks. Following this, significance of genes is propagated in the context of global heterogeneous network of biological concepts from independent categories until convergence is observed.

In the first step to prioritize genes in local bipartite, in order to perform eigenvector-based random walk heuristics such as PageRank ([3](#_ENREF_3)), HITS ([4](#_ENREF_4)) and SALSA ([5](#_ENREF_5)), edges connecting vertices are bi-directional. Since previous studies have suggested SALSA outperformed competitors in several contexts ([6](#_ENREF_6)), we adopted SALSA to prioritize mRNAs in the local bipartite graphs.

To carry this out on the test set gene feature bipartite matrix, let $G=(V, E)$ denote the graph, where $V$ is the set of vertices and $E$ is the set of edges. The adjacency matrix $W$ of the graph $G$ is derived by scoring $W\left[ i, j \right]=1$ if there is a link from node $i$ to $j$ and is the input to each of the link-analysis algorithms. For each node $i$, let $B\left( i \right)= \left\{ j: W\left[ i, j \right]=1 \right\}$ denotes the backward neighbors of node $i$, $F\left( i \right)= \left\{ j: W\left[ j, i \right] \right\}=1$ as its forward neighbors and $N\left( i \right)$ as the neighboring set. Thus, $N\left( i \right)=B\left( i \right) \bigcup F\left( i \right)$. We use matrix $A$ to denote the authority scores of nodes; $H$ to denote the hub scores; and $S$ to denote the significance of each node.

SALSA algorithm, which can be viewed as a combination of PageRank and HITS, splits the set of vertices into a bipartite graph and then performs a random walk alternating between the hubs and authority sides. Each vertex divides its authority/hub weight equally among the nodes connected to it. That is,

$a_{i} \leftarrow\sum_{j :W_{\mathrm{ji}}=1} {\frac{1}{\left| F\left( j \right) \right|}h}_{j}$ (1)

$h_{i} \leftarrow\sum_{j :W_{\mathrm{ij}}=1} {\frac{1}{\left| B\left( j \right) \right|}a}_{j}$ (2)

Eventually the two scores are combined to generate the significance score of each vertex.

Weights can then be computed explicitly without the iterative process and there is rapid convergence to exact values. In our experiments using sets of 500 genes and/or their annotations, most convergences were reached within 25 iterations.

Since each homo-subnetworks of mRNAs and biological concepts can be viewed as bipartites with bi-directional edges, significance score for each mRNA can be updated iteratively as:

$s_{i}\overset{\leftarrow}{}\sum_{j :j \in N(i)} \frac{1}{\left| N\left( j \right) \right|}s_{j}$ (3)

where $s_{i}$ is the significance score of node $i$ and $N(i)$ are the neighbors of node $i$.

The task to prioritize genes in the context of heterogeneous networks requires similar techniques to propagate significance scores from subnetworks. However, unlike other cases, here we are attempting to compute the significance of nodes on one side of the bipartites (genes), while leaving the significance scores of the nodes on the other side unchanged (annotations). Thus, an intuitive and straightforward idea is to conceive a schema that allows all significance scores from different features to contribute to the global significance at a fractional rate $\mu$. To do this, let $\mu$ be a constant where $0<\mu<1$. For each iteration, at each step $t$ for feature $i$, a gene’s significance score $Sig$ is updated,

$\mathrm{Sig}_{t}= \mu\mathrm{Sig}_{t-1}+ \left( 1-\mu\right) \mathrm{score}_{i}$ (4)

All scores from each feature contribute to the significance score at each round, and a small constant $\varepsilon$ $\left( 0<\varepsilon\right)$ is defined as the convergence marker. In our experiments we saw good performance against known targets when $0.6<\mu<0.8$ and the convergence marker $\varepsilon$ from 0.95 to 0.99. The convergence was proved by Zhou *et al*. and the final iteration vector was independent from the initial starting value ([7](#_ENREF_7)).

**REFERENCES**

1. Oğul, H. (2014), *miRNomics: MicroRNA Biology and Computational Analysis*. Springer, pp. 243-256.

2. Hwang, T. and Kuang, R. (2010) A Heterogeneous Label Propagation Algorithm for Disease Gene Discovery. *SIAM '2010: Proceedings of SIAM International Conference on Data Mining (SDM)*, 583-594.

3. Page, L., Brin, S., Motwani, R. and Winograd, T. (1998).

4. Kleinberg, J. (1999) Hubs, authorities, and communities. *ACM Comput. Surv.*, **31**, 5.

5. Lempel, R. and Moran, S. (2000), *ACM Transactions on Information Systems*, Vol. 19, pp. 387-401.

6. Farahat, A., Lofaro, T., Miller, J., Rae, G. and Ward, L. (2006) Authority Rankings from HITS, PageRank, and SALSA: Existence, Uniqueness, and Effect of Initialization. *SIAM J. Sci. Comput.*, **27**, 1181-1201.

7. Zhou, D., Bousquet, O., Lal, T.N., Weston, J. and Schölkopf, B. (2004) Learning with local and global consistency. *Advances in neural information processing systems*, **16**, 321-328.
